# Development of Liver-Targeting α_V_β_5_^+^ Exosomes as Anti-TGF-β Nanocarriers for the Treatment of the Pre-Metastatic Niche

**DOI:** 10.3390/biology13121066

**Published:** 2024-12-19

**Authors:** Paloma Acosta Montaño, Eréndira Olvera Félix, Veronica Castro Flores, Arturo Hernández García, Ruben D. Cadena-Nava, Octavio Galindo Hernández, Patricia Juárez, Pierrick G. J. Fournier

**Affiliations:** 1 Posgrado en Ciencias de la Vida, Centro de Investigación Científica y de Educación Superior de Ensenada (CICESE), Ensenada 22860, BC, Mexico; acostabp@cicese.edu.mx (P.A.M.); erendira@cicese.edu.mx (E.O.F.); vecaflo.qfb@gmail.com (V.C.F.); hernandez.arturo@uabc.edu.mx (A.H.G.); 2 Departamento de Innovación Biomédica, Centro de Investigación Científica y de Educación Superior de Ensenada (CICESE), Ensenada 22860, BC, Mexico; pjuarez@cicese.mx; 3 Centro de Nanociencias y Nanotecnología, Universidad Nacional Autónoma de México (UNAM), Ensenada 22860, BC, Mexico; rcadena@ens.cnyn.unam.mx; 4 Laboratorio Multidisciplinario de Estudios Metabólicos y Cáncer, Facultad de Medicina Mexicali, Universidad Autónoma de Baja California (UABC), Mexicali 21000, BC, Mexico; octavio.galindo@uabc.edu.mx

**Keywords:** exosome, drug delivery, targeted therapy, integrin, TGF-β, cancer, pre-metastatic niche

## Abstract

Liver metastases frequently occur in pancreatic and colorectal cancer. Their development is promoted by tumor-derived exosomes with the integrin α_V_β_5_ on their membrane. This integrin directs exosomes to the liver, where they promote a TGF-β-dependent pre-metastatic niche. We proposed the development of α_V_β_5_^+^ exosomes to deliver anti-TGF-β therapy to the liver. This study demonstrates that the overexpression of α_V_β_5_ in 293T cells allows its transfer to the secreted exosomes. α_V_β_5_ overexpression increases exosome delivery to the liver, and α_V_β_5_^+^ exosomes accumulate more in the liver compared to the lungs, kidneys, and brain in mice. We then sought 293T cells to directly produce and load an anti-TGF-β agent in their exosomes. First, we transduced 293T cells to express shRNAs against *Tgfb1*; however, the exosomes isolated from these cells did not knock down *Tgfb1* in treated macrophages in vitro. However, when 293T expressed an mRNA coding a soluble form of betaglycan (sBG), a TGF-β inhibitor, this mRNA was detected in the isolated exosomes and the protein in the conditioned media of macrophages treated in vitro. In turn, this conditioned media decreased the TGF-β-induced phosphorylation of SMAD2/3 in hepatic cells in vitro. Our findings suggest that α_V_β_5_^+^ exosomes could serve as nanocarriers for liver-targeted anti-TGF-β therapies.

## Simple Summary

Exosomes are small vesicles released by our cells to communicate. Tumor cells also release exosomes that can go to the liver when they have the integrin α_V_β_5_, an adhesion receptor, on their surface. These exosomes reach the liver first, where their content establishes a pre-metastatic niche that increases the chances of the upcoming metastatic cells surviving and forming a liver metastasis, a frequent complication in pancreatic and colorectal cancer. To prevent this pre-metastatic niche in the liver, we were inspired by cancer cells and modified some non-cancerous cells to produce more integrin α_V_β_5_. Consequently, this integrin was transferred to its exosomes, and the exosomes we collected accumulated more efficiently and specifically in the liver of mice. Since the protein TGF-β initiates the pre-metastatic niche, we further modified our cells to produce the mRNA of a TGF-β inhibitor. Cells that internalized the exosomes containing these mRNA produced the TGF-β inhibitor and inhibited TGF-β signaling. These functionalized exosomes could then be a nanocarrier to bring TGF-β inhibitors specifically to the liver to limit their side effects and prevent the pre-metastatic niche.

## 1. Introduction

The liver is a common site for metastasis in colorectal and pancreatic cancer [1]. Establishing a pre-metastatic niche facilitates the development of metastases, including in the liver, by promoting the colonization of the new organ [2]. Factors secreted by the primary tumor promote the establishment of this pre-metastatic niche, such as exosomes, small extracellular vesicles ranging from 40 to 160 nm in diameter [3,4]. Exosomes derived from pancreatic cancer cells tend to accumulate in the liver, where they are internalized in Kupffer cells, liver-residing macrophages, increasing their production of transforming growth factor β (TGF-β) [5]. This TGF-β, in turn, triggers hepatic stellate cells to produce fibronectin deposits, facilitating the recruitment of bone-marrow-derived cells and establishing a platform for the incoming metastatic cells. The inhibition of TGF-β signaling with an inhibitor of the TGF-β type 1 receptor (TGFBR1) prevented the formation of this pre-metastatic niche and pancreatic cancer metastasis to the liver in a mouse model [5].

TGF-β is a well-established metastasis promoter that stimulates the epithelial-to-mesenchymal transition, invasion, and colonization, making it a promising target in cancer [6]. Different anti-TGF-β drugs have been developed and tested, including TGF-β neutralizing agents like antibodies and soluble forms of TGFBR2 (TGFBR2-Fc) or TGFBR3, also known as betaglycan (BG). TGFBR3 binds to TGF-β to facilitate its presentation to TGFBR2, initiating the signaling cascade. However, when its extracellular domain undergoes proteolytic cleavage, it can neutralize TGF-β. Treatment with soluble BG (sBG) to sequester TGF-β inhibits cancer cell growth, angiogenesis, and metastasis in vivo [7,8]. However, systemic inhibitors of TGF-β that have progressed to clinical trials have demonstrated either limited efficacy or significant adverse effects in patients, highlighting the critical role of TGF-β in normal physiology, indicating the necessity of the site-specific delivery of these inhibitors.

Exosomes facilitate intercellular communication and modulate local or distant microenvironments by the horizontal transfer of their cargo to the recipient cells [9]. They can also be functionalized and loaded with therapeutic agents, such as inhibitory molecules or small interfering RNA (siRNA), effectively inhibiting gene expression in targeted cells [10]. Due to these characteristics, the use of exosomes as a drug delivery system has emerged in recent years [11]. Genetically engineered 293T-cell-derived exosomes have emerged as a promising strategy to improve targeted therapy and attenuate the secondary effects of cancer treatment. Nonetheless, their distribution inside the body is not random. As described by Hoshino et al. [12], specific integrins promote distribution to particular organs and exosomes from cancer cells with the integrin α_V_β_5_ accumulating in the liver.

In this study, we proposed developing a liver-targeted anti-TGF-β treatment using functionalized α_V_β_5_^+^ exosomes. By overexpressing the integrin α_V_β_5_ in 293T cells, we enhanced the ability of their exosomes to accumulate in the liver of mice. To load these exosomes with an anti-TGF-β agent, we transduced 293T cells to express either a short hairpin RNA (shRNA) against *Tgfb1* to knock it down in cells internalizing the exosomes or an mRNA coding for sBG, enabling cells that internalized the exosomes to secrete this TGF-β neutralizing agent. Although the exosomes derived from 293T-shTgfb1^+^ cells did not affect *Tgbf1* levels, exosomes from 293T cells expressing secreted sBG in the conditioned media caused a decrease in SMAD2/3 phosphorylation. In summary, our study offers insight into the use of α_V_β_5_^+^ exosomes as a novel nanocarrier to target the liver, taking advantage of the cargo-carrying capability of these exosomes to deliver anti-TGF-β agents and prevent toxic effects in patients.

## 2. Materials and Methods

### 2.1. Cell Lines and Cell Culture

Human embryonic kidney cells (293T (CRL-3216)), mouse macrophage cells (RAW 264.7 (TIB-71)), human hepatocarcinoma cells (HepG2 (HB-8065)), and mouse breast cancer cells (4T1 (CRL-2539)) were obtained from the American Type Culture Collection (ATCC, Manassas, VA, USA). The 293T cells were cultured in high-glucose Dulbecco’s modified Eagle medium (DMEM, Corning, Corning, NY, USA). The RAW 264.7 and 4T1 cells were cultured in Roswell Park Memorial Institute (RPMI) 1640 media (Corning), and HepG2 cells were cultured in Eagle’s Minimum Essential Medium (EMEM, Corning). All media were supplemented with 10% (*v*/*v*) heat-inactivated fetal bovine serum (FBS; Atlanta Biologicals, Flowery Branch, GA, USA or Biowest USA, Riverside, MO, USA) and 1% (*v*/*v*) antibiotic/antimycotic solution (Corning). Cells were maintained at 37 °C with 5% CO_2_ in a humidified chamber. Exosome-depleted FBS was obtained by ultracentrifugation for 16 h using an SW 32 Ti rotor in an Optima XPN-100 ultracentrifuge (Beckman Coulter, Brea, CA, USA), followed by filtration with a 0.2 µm filter.

### 2.2. Exosome Preparation

Exosomes were isolated using differential centrifugation of conditioned media [13]. 293T cells were cultured in 150 mm cell culture dishes until reaching 80% confluence and further cultured in a media containing 2% (*v*/*v*) exosome-depleted FBS for 48 h. The conditioned media were collected and subsequently subjected to sequential centrifugation: 300× *g* for 10 min, 2000× *g* for 20 min, and 10,000× *g* for 30 min at 4 °C. The supernatant was stored at −80 °C or ultracentrifuged at 120,000× *g* using a 50.2 Ti rotor (Beckman Coulter) for 2 h at 4 °C. Pellets were resuspended in filtered PBS 1× and ultracentrifuged at 120,000 g using a 90 Ti rotor for 1.5 h at 4 °C or labeled using SP-DiIC_18_ (Thermo Fisher Scientific, Waltham, MA, USA). SP-DiIC_18_ (2 mM in DMSO) was added to the exosome solution (with final concentration of 1.0–5.0 µM), and the solution was incubated for 40 min at 37 °C, followed by 15 min on ice. Exosomes were washed in PBS 1× and ultracentrifuged (120,000× *g* in a 90 Ti rotor for 1.5 h at 4 °C) to eliminate excess dye. For the negative controls, we mixed PBS 1× with SP-DiIC_18_ at the same concentration (1.0–5.0 µM), and the same process was applied as for the exosomes. Exosomes were resuspended in filtered PBS 1×, and protein concentration was measured using the Micro BCA Protein Assay Kit (Thermo Fisher Scientific). The purified exosomes were then characterized or immediately used for functional assays (e.g., cellular uptake in vitro and distribution in vivo).

### 2.3. Characterization of Exosome Size

We used dynamic light scattering (DLS) and transmission electron microscopy (TEM) to determine the size and morphology of the exosomes prepared. For DLS, exosome solutions were transferred to a ZEN40 cuvette (Malvern Panalytical, Malvern, UK), and the hydrodynamic diameter was measured using a Zetasizer Nano ZS (Malvern Panalytical) with PBS 1× as a dispersant. Each sample was measured in triplicate with 15 individual measurements each. For TEM, samples were placed on a carbon-coated copper grid (400 mesh, Ted Pella, Redding, CA, USA) and incubated for 5 min. Excess liquid was removed with filter paper, and samples were stained with uranyl acetate (2% *w*/*v*) for 7 min. Grids were dried with filter paper, and samples were then analyzed using a transmission electron microscope (H7500, Hitachi, Hatoyama, Japan) at a voltage of 80 kV. The digital images were analyzed using FIJI software v1.54 or higher [14], and the diameter of 89 exosomes was measured.

### 2.4. In Vitro Cellular Uptake Assay

For exosome internalization assays, RAW 264.7 cells were seeded at a density of 120,000 cells/cm^2^. The next day, exosomes were added (0.75–6.0 µg/cm^2^), and the cells were cultured for an additional 24 h. Cells were fixed with paraformaldehyde for fluorescence microscopy and counterstained with DAPI (0.1 μg/mL, Thermo Fisher Scientific) overnight. Fluorescent images were obtained using the Floid Cell Imaging System (Thermo Fisher Scientific) and analyzed using FIJI software v1.54 or higher [14]. For flow cytometry analysis and western blot, cells were analyzed as described below.

### 2.5. Distribution of Exosomes in Vivo

Animal protocols were performed following the Federal Regulation for Animal Experimentation and Care (SAGARPA, NOM-062-ZOO, 1999, Mexico City, Mexico) at the Center of Scientific Research and Higher Education of Ensenada (CICESE). Balb/C (strain Balb/CAnNHsd) mice were obtained from Envigo (Mexico City, Mexico) and bred at the CICESE. Mice received water and food (Laboratory Autoclavable Rodent Diet, 24% protein content; LabDiet, St. Louis, MO, USA) ad libitum and were housed in individually ventilated cages (Optimice System, Animal Care Systems, Centennial, CO, USA) in a 12 h light/12 h dark cycle at 20–24 °C.

Mice (male, 6–8 weeks old) received an intravenous inoculation of PBS 1× or exosomes (10–40 μg of total protein) via the tail vein in a total volume of 200 μL using a 500 µL syringe with a 30 G needle. The next day (16–24 h later), the mice were euthanized. Organs were collected and embedded in the Tissue-Tek O.C.T. compound (Sakura Finetek, Torrance, CA, USA), and blocks were frozen over dry ice. Sections (8 µm thick) were prepared using a CM1510S cryostat (Leica, Wetzlar, Germany), air-dried, fixed with paraformaldehyde (2% *v*/*v*), and counterstained using DAPI (1 μg/mL). Fluorescent images were obtained using an FV1000 Olympus confocal microscope (Evident, Tokyo, Japan) and analyzed using FIJI software v1.54 or higher [14].

### 2.6. Flow Cytometry Analysis

RAW 264.7 cells were directly analyzed using flow cytometry in exosome uptake experiments. 293T cells were stained using fluorescent antibodies before the analysis. Trypsinized 293T cells were resuspended in a blocking buffer (FBS 10% *v*/*v*, BSA 1% *w*/*v*, EDTA 0.5 mM, PBS 1× pH 7.4) for 10 min at room temperature. Cells were stained with a PE-conjugated antibody against α_V_β_5_ (125 ng/10^6^ cells, clone P1F6, Biolegend, San Diego, CA, USA), α_V_ (62.5 ng/10^6^ cells, clone NKI-M9, Biolegend), or β_5_ (62.5 ng/10^6^ cells, clone AST-3T, Biolegend) and incubated at 4 °C for 40 min in the dark. Cells were washed and resuspended with FACS buffer (BSA 1% *w*/*v*, EDTA 0.5 mM, PBS 1×, pH 7.4) and analyzed immediately after using an Attune acoustic focusing cytometer (Applied Biosystems, Foster City, CA, USA). Samples were analyzed using the Attune software (v2.1; Applied Biosystems). Singlets were gated using forward scatter area (FSC-A) versus height (FSC-H) and side scatter area (SSC-A) versus height (SSC-H) density plots, and cells were gated using FSC-A versus SSC-A density plots. Unstained 293T cells were used to establish basal fluorescence. Flow cytometry data were analyzed using the Attune software (v2.1).

### 2.7. Western Blotting

For whole cell protein extraction, cells were detached, centrifuged (800× *g*, 5 min, 4 °C), and resuspended in a lysis buffer (NP-40 1% *v*/*v*, NaCl 150 mM, Tris-HCl 50 mM, pH 8.0) supplemented with cOmplete Mini EDTA-free (Roche Applied Science, Penzberg, Germany) and ETDA (1 mM) protease inhibitors. To assess SMAD2/3 phosphorylation, the lysis buffer also contained SDS (0.1 *w*/*v*) and the phosphatase inhibitor PhoSTOP (Roche Applied Science). After 10 min of incubation on ice, lysates were centrifuged to remove cell debris (14,000× *g*, 10 min, 4 °C). Protein concentration was measured using the Micro BCA Protein Assay Kit (Thermo Fisher Scientific). Cell lysates (25–30 µg of protein) or exosome preparations (5 µg of protein) were mixed with 5× Laemmli buffer and incubated at 95 °C for 5 min. Proteins were separated using SDS-PAGE and transferred onto Immobilon-P PVDF membranes (Merck Millipore, Darmstadt, Germany) using a semi-dry transfer system (Bio-Rad, Hercules, CA, USA). After transfer, membranes were stained with a Ponceau S red solution to confirm protein transfer and were blocked in a 1× TBS solution containing Tween 20 (0.1% *v*/*v*) and non-fat dry milk (5% *w*/*v*) for 1 h at room temperature. Membranes were then incubated overnight at 16 °C in the blocking solution (TBS-T-Milk) containing a primary antibody against CD81 (dilution 1:250 or 0.8 µg/mL, mouse monoclonal IgG_2b_ κ, clone B-11, Santa Cruz Biotechnology, Dallas, TX, USA), T7 (dilution 1:2500 or 0.4 µg/mL, rabbit polyclonal, Merck Millipore), HA (dilution 1:200 or 1.0 µg/mL, mouse monoclonal, IgG_2a_ κ, clone F-7, Santa Cruz Biotechnology), Myc (dilution 1:1000 or 0.2 µg/mL, mouse monoclonal IgG_1_, clone 9E10, Santa Cruz Biotechnology), phospho-SMAD2/3 (dilution 1:1000, rabbit polyclonal, Cell Signaling, Danvers, MA, USA), SMAD2/3 (dilution 1:1000, rabbit polyclonal, Cell Signaling), or α-tubulin (dilution 1:8000 or 0.625 µg/mL, mouse monoclonal IgG_1_ κ, clone B-5-1-2, Sigma-Aldrich, St. Louis, MO, USA). Membranes were washed thrice in TBS-T and incubated with anti-rabbit IgG (dilution 1:80,000 or 0.09 µg/mL, Sigma-Aldrich) or anti-mouse IgG (dilution 1:40,000 or 0.19 µg/mL, Sigma-Aldrich) secondary antibodies conjugated to horseradish peroxidase for 1 h at room temperature. Protein detection was performed using the Immobilon Western Chemiluminescent HRP substrate (Merck Millipore) and a ChemiDoc XRS (Bio-Rad).

### 2.8. Plasmid Preparation

For the overexpression of the human subunit β_5_, we used the pCX-EGFP beta5 integrin receptor plasmid (a gift from Raymond Birge, Addgene #14996) [15]. The coding sequence of the human ITGB5 (Table 1) was amplified using the Q5 High-Fidelity DNA Polymerase (New England Biolabs, Ipswich, MA, USA), adding a T7 tag to the 3′ extremity, and the PCR product was purified using GeneJET PCR Purification Kit (Thermo Fisher Scientific). The pLenti CMV GFP Hygro (656-4) plasmid (a gift from Eric Campeau and Paul Kaufman, Addgene #17446) [16] was linearized using XbaI and SalI restriction enzymes (New England Biolabs) ran on an agarose gel, and the linearized plasmid was purified using the GenElute Gel Extraction Kit (Sigma-Aldrich). The ITGB5 coding sequence was inserted in the linearized pLenti-CMV Hygro vector using the Gibson Assembly Cloning kit (New England Biolabs) to obtain the pLenti-ITGB5-T7-Hygro plasmid.

For the expression of a soluble form of rat betaglycan (sBG), we used pCMV5-sBG-myc (a gift from Fernando López-Casillas, UNAM, Mexico City, Mexico) [17]. The coding sequence was amplified using the Q5 High-Fidelity DNA Polymerase (New England Biolabs), and the PCR product was purified using the GeneJET PCR Purification Kit (Thermo Scientific). The pLJM1 eGFP plasmid (a gift from David Sabatini, Addgene plasmid #19319) [18] was linearized with NheI and EcoRI restriction enzymes (New England Biolabs), run on an agarose gel, and the pLJM1 backbone was purified using the GenElute Gel Extraction Kit (Sigma-Aldrich). The sBG amplification product was inserted in the linearized pLJM1 vector using the Gibson Assembly Cloning kit (New England Biolabs) to obtain the pLJM1-sBG-myc plasmid. A sequence reported to direct mRNA to exosomes or exosome zipcode, ACCCTGCCGCCTGGACTCCGCCTGT, was inserted in the 3′ UTR, 18 nucleotides before and/or 22 nucleotides after the cPPT/CTS region of the pLJM1-sBG-myc plasmid using Q5 Site-Directed Mutagenesis Kit (New England Biolabs). The oligonucleotides used are listed in Table 1.

For the knockdown of mouse *Tgbf1*, we used two different shRNA recognition target sequences: GCTCTTGTGACAGCAAAGATA (shTgfb1^1^, corresponding to the clone TRCN0000065997 from the TRC library (21)) and GTGGAACTCTACCAGAAATAT (shTgfb1^2^, using the VectorBuilder’s shRNA Target Design tool, Chicago, IL, USA). The sequences for the corresponding shRNA were inserted right after the AgeI restriction site of the pLKO.1 puro plasmid (a gift from Bob Weinberg, Addgene plasmid #8453) [19] using Q5 Site-Directed Mutagenesis Kit (New England Biolabs). The oligonucleotides used are listed in Table 1.

Newly obtained plasmids were transformed into chemically competent *E. coli* NEB5α, and colony PCR was used to confirm insertion (Table 2). For plasmid purification, single colonies were picked and inoculated in 10 mL of LB medium supplemented with carbenicillin (100 μg/mL) and incubated overnight at 37 °C while shaking at 200 rpm. Bacteria cultures were harvested by centrifugation, and plasmid DNA was extracted using the GeneJet Plasmid Miniprep Kit (Thermo Fisher Scientific). Plasmid concentration was measured using Nanodrop Lite (Thermo Fisher Scientific). Purified plasmids were sequenced using Sanger sequencing (Genewiz, NJ, USA).

### 2.9. Transfection and Lentiviral Transduction

For the overexpression of α_V_β_5_ in 293T cells, we transfected cells with pCMV3-ITGAV-HA (Sino Biological, Beijing, China) and pLenti-ITGB5-T7 using the calcium phosphate protocol. Cells were seeded one day before transfection. Plasmids were diluted to 12.5 ng/μL in a CaCl_2_ solution (240 mM), and this solution was added to an HEPES buffer saline solution (1.5 mM Na_2_HPO_4_, 280 mM NaCl, HEPES, 50 mM) of equal volume. The transfection mix (equivalent to 250 ng/cm^2^ per plasmid) was added to cultured 293T cells, and cell culture media were changed the next day. Protein expression was confirmed by flow cytometry three days after the transfection, or cells were selected with 100 µg/mL of hygromycin (Gold Bio, St. Louis, MO, USA).

For the overexpression of sBG, eGFP, shTgfb1, or shGFP in 293T cells, we used a third-generation lentiviral system consisting of the packaging vectors pLP1 and pLP2 (Thermo Fisher Scientific), the envelope vector pMD2.G (a gift from Didier Trono, Addgene plasmid #12259), and the transfer vectors pLJM1-sBG-myc, pLenti-CMV-GFP-puro (658-5) (Addgene plasmid #17448) [16], pLKO.1-shTgfb1, pLKO.1-GFP-shRNA, and pLKO.1-puro (Addgene plasmid #30323) [18]. Equimolar quantities of plasmids were transfected in 293T cells using a calcium phosphate protocol. Lentiviral particles were harvested 48 and 72 h after the transfection, pooled, and supplemented with polybrene (8 µg/mL, Sigma-Aldrich) before their transduction into 293T-α_V_β_5_ or 4T1 cells. Transduced cells were selected using 0.5 or 5.0 µg/mL of puromycin (Corning) for 293T-α_V_β_5_ or 4T1 cells, respectively.

### 2.10. Tgfb1 Silencing and Neutralization Assays

For shRNA validation, shRNA plasmids were transduced into 4T1 or 293T-α_V_β_5_ cells and selected with 0.5 μg/mL of puromycin. Cells were pelleted for RNA extraction. For shRNA-mediated knockdown of TGF-β1, exosomes from 293T-α_V_β_5_, 293T-α_V_β_5_-shGFP, 293T-α_V_β_5_-shTgfb1^1^, 293T-α_V_β_5_-shTgfb1^2^, and 293T-α_V_β_5_-shTgfb1^1&2^ were obtained by ultracentrifugation. RAW 264.7 cells, seeded at 120,000 cells/cm^2^, were treated the next day with 6.0 μg/cm^2^ of exosomes in RPMI supplemented with 2% (*v*/*v*) FBS and LPS from *E. coli* o26:B6 (500 ng/mL, Sigma-Aldrich) for 8, 12, or 24 h before RNA extraction from cells or collection of the conditioned media. For exosome-delivered sBG mRNA, exosomes were obtained from 293T-sBG-Zip^1^ cells. RAW 264.7 cells, seeded at 120,000 cells/cm^2^, were treated the next day with 6.0 μg/cm^2^ of exosomes in RPMI supplemented with 2% (*v*/*v*) FBS and LPS from *E. coli* o26:B6 (500 ng/mL, Sigma-Aldrich) for 24 h. The conditioned media of RAW cells were used to treat HepG2 for 2 h before obtaining protein lysates.

### 2.11. Quantitative Real-Time PCR

Total RNA was extracted from 4T1 or RAW 264.7 cells or exosomes using the GeneJET RNA Purification Kit (Thermo Fisher Scientific) according to the manufacturer’s protocol. RNA was reversed-transcribed using anchored Oligo dT primers (Thermo Fisher Scientific) and SuperScript™ II reverse transcriptase (Thermo Fisher Scientific). Complementary DNA (cDNA) was analyzed in triplicate by quantitative real-time PCR using SYBR™ Green PCR Master Mix (Thermo Fisher Scientific, Waltham, MA, USA) or QuantiTect SYBR^®^Green Master Mix (Qiagen, Hilden, Germany) for 40 cycles (95 °C for 15 s; 58 °C for 30 s; 72 °C for 30 s) using a 7500 Real-Time PCR System (Thermo Fisher Scientific). Quantification of *sBG* expression was performed using standard curves of diluted cDNA templates, and relative amounts were normalized by using human *RPL32* as a reference gene, while for *Tgfb1* gene expression, mouse *Rpl32* was used as a reference gene. Primer sequences (T4Oligo, Irapuato, Mexico) are listed in Table 2.

### 2.12. Statistical Analysis

Statistical analyses were performed using Prism v9 or higher (GraphPad Software, Inc., La Jolla, CA, USA). Tests for normal (Gaussian) distribution were performed using the Shapiro–Wilk test. Comparisons of the two groups were conducted using a two-tailed Student’s *t*-test. Comparisons for three or more groups were conducted using a 1-way analysis of variance (ANOVA) test, followed by Tukey’s post hoc test. For responses that were affected by two variables, a 2-way ANOVA with Tukey’s post hoc test was used. Results are expressed as the mean  ±  SEM or presented as box plots with median, interquartile range, and all data points. A *p*-value inferior to or equal to 0.05 (*p* ≤ 0.05) was considered significantly different.

## 3. Results

### 3.1. Exosomes from 293T Cells Accumulate in the Liver

**Figure 1:**
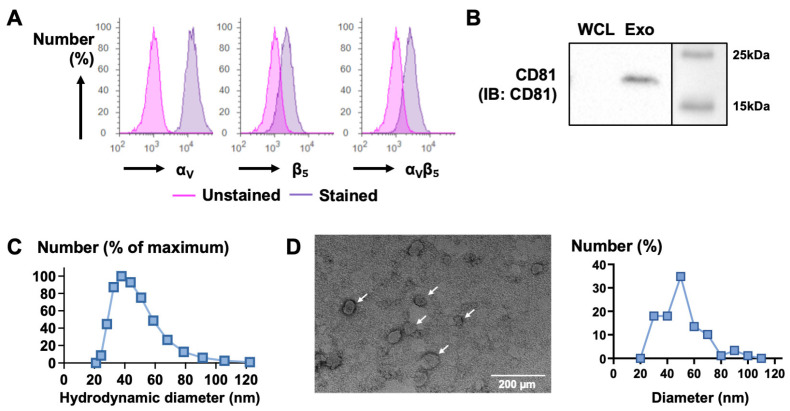
Integrin expression in 293T cells and validation of isolated exosomes. (**A**) Expression of the integrins α_v_ (**left**), β_5_ (**middle**), and α_v_β_5_ (**right**) in 293T cells using flow cytometry. Results are presented as histograms. (**B**) Western blot analysis of the exosomal marker CD81 in whole cell lysates (WCL) and exosomes (Exo) from 293T cells. (**C**) Measurement of the hydrodynamic diameter of the exosomes isolated from 293T cells. Results are presented as a histogram of the particle size distribution. (**D**) Characterization of the exosome morphology and size using TEM. Results are presented as a representative image (**left**; arrows indicate the exosomes) and a histogram (**right**) of the exosome size distribution.

As a source of exosomes, we sought a cell line that (1) can be easily engineered by transfection or transduction, (2) produces exosomes, (3) is non-cancerous to avoid the risk of establishing a pre-metastatic niche, and (4) expresses or can express the integrin α_V_β_5_ for accumulation in the liver. Taking into account the first three criteria and previous publications [20,21,22], we selected the human embryonic kidney 293T cell line and sought to determine whether it expresses the integrin α_V_β_5_. Flow cytometry detected the presence of both subunits α_V_ and β_5_ and the dimer α_V_β_5_, indicating a basal level of expression of the integrin α_V_β_5_ on the membrane of 293T cells (Figure 1A). Next, we used a differential centrifugation protocol [23] to isolate exosomes from the conditioned media of 293T cells and characterize the vesicles obtained. CD81, a member of the tetraspanin family and an exosome marker, was not detected in the whole cell lysate but was detected in the exosomal fraction using a western blot (Figure 1B). We then assessed the hydrodynamic diameter of the vesicles obtained using dynamic light scattering. Their diameter ranged from 21.0 to 122.4 nm, with an average of 39.7 ± 16.0 nm (Figure 1C). Transmission electron microscopy showed vesicles with a sphere-shaped morphology and a diameter ranging from 26.8 to 101.0 nm, with an average of 50.5 ± 15.4 nm (Figure 1D). Considering their shape, diameter, and the presence of the exosome marker CD81, these results confirmed that the vesicles isolated from 293T cells are indeed exosomes [24].

**Figure 2:**
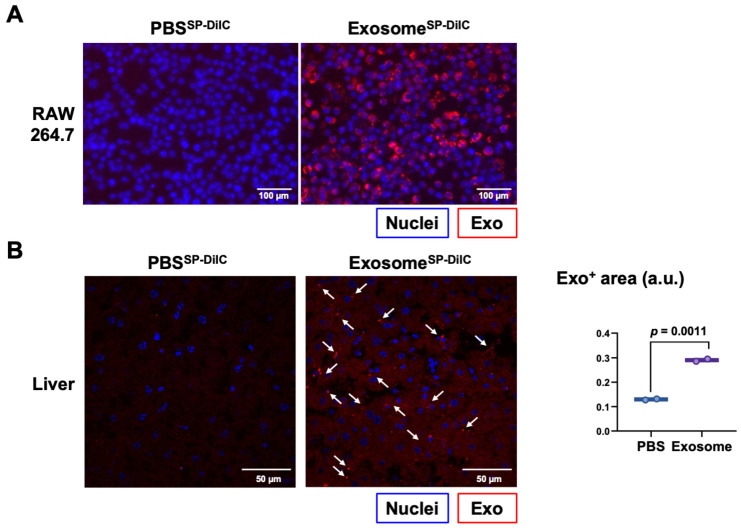
Exosomes from 293T cells localized to the liver. (**A**) Fluorescence microscopy of RAW 264.7 cells cultured for 24 h with a control solution (PBS^SP-DiIC^) or 293T cell-derived exosomes (3 μg/cm^2^) stained with SP-DiIC_18_ (Exosome^SP-DiIC^) (2.5 µM SP-DiIC_18_). (**B**) Confocal microscopy of liver sections from mice inoculated with a control solution (PBS^SP-DiIC^) or SP-DiIC_18_-stained exosomes from 293T cells (Exosome^SP-DiIC^) (5.0 µM SP-DiIC_18_). Results are presented as representative images of liver sections (**left**, arrows indicate exosome foci) and the quantification (**right**) of exosome-positive (Exo^+^) areas by fluorescence imaging in arbitrary units (a.u.) (*N* = 2 per group) and compared using an unpaired Student’s *t*-test.

To determine if macrophage-like cells can internalize these exosomes, we labeled exosomes isolated from 293T cells with a fluorescent membrane probe, SP-DiIC_18_. The fluorescently labeled exosomes were then cultured with RAW 264.7 cells, a mouse macrophage-like cell line. Twenty-four hours later, we confirmed the presence of the fluorescent exosomes in the RAW 264.7 cells (Figure 2A). To determine whether exosomes from 293T cells with an endogenous expression of the integrin α_V_β_5_ can target the liver, Balb/C mice were injected intravenously with a control solution (PBS^SP-DiIC^) or fluorescently labeled exosomes, and 24 h later, their livers were collected. Confocal microscopy indicated the presence of fluorescence in the livers of the mice inoculated with the exosomes (Figure 2B). These results confirm the uptake of exosomes from 293T cells in macrophages in vitro and their capacity to accumulate in the liver in vivo, although we could not confirm whether the uptake in the livers was in Kupffer cells.

### 3.2. Overexpression of the Integrin α_V_β_5_ Increases Exosome Accumulation in the Liver

Although the exosomes from the 293T cells were detected in the livers, we sought to increase their accumulation in the livers, considering that the levels of the integrin α_V_β_5_ were relatively low, possibly due to the lower expression of the β_5_ subunit compared to the α_V_ subunit (Figure 1A). Thus, we decided to engineer the 293T cells to overexpress the subunits α_V_ and β_5_. We co-transfected 293T cells with plasmids containing the coding sequence of the human HA-tagged α_V_ (ITGAV-HA) and T7-tagged β_5_ (ITGB5-T7) subunits.

**Figure 3:**
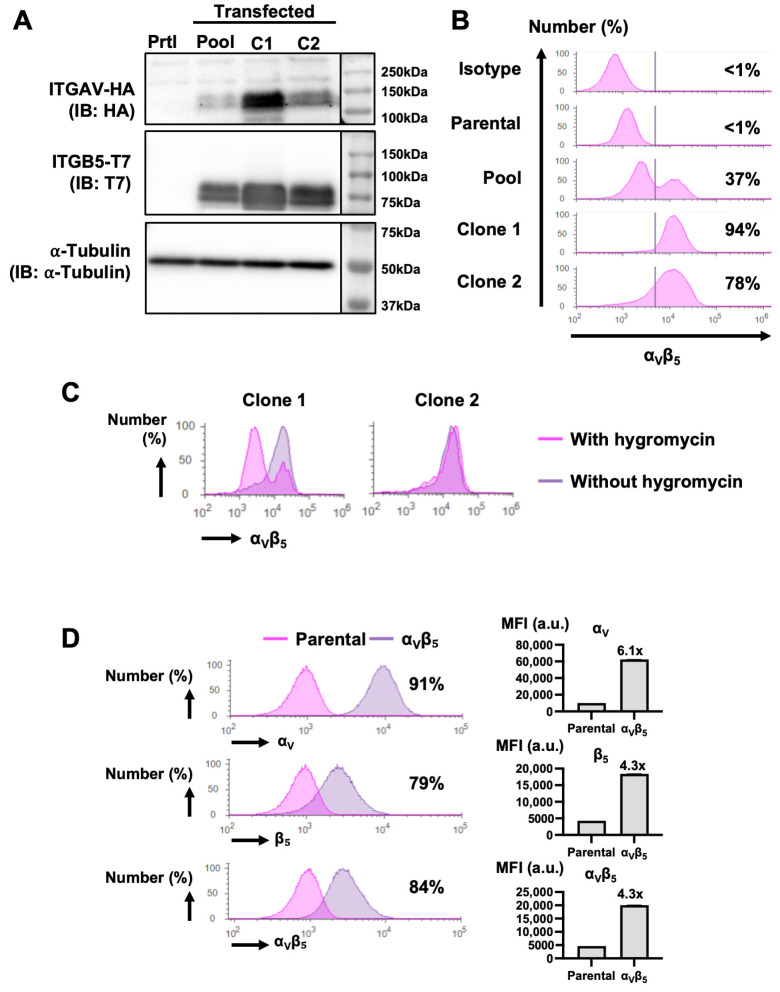
Modification and selection of 293T cells overexpressing the integrin α_v_β_5_. (**A**) Western blot analysis of the expression of the exogenous integrin subunits ITGAV-HA and ITGB5-T7 in untransfected 293T cells (Prtl), after transfection and antibiotic selection (Pool), or after selection of 2 clones, clone 1 (C1) and clone 2 (C2). α-tubulin was used as a loading control. (**B**) Flow cytometry analysis of the basal fluorescence of 293T cells (Isotype), the expression of α_v_β_5_ in untransfected cells (Parental), after transfection and antibiotic selection (Pool), and after selection of clones using limited dilution (clone 1 and clone 2). Results are presented as histograms, with the number indicating the percentage of cells overexpressing α_v_β_5_. (**C**) Flow cytometry analysis of α_v_β_5_ expression in clone 1 and clone 2 after being cultured for 2 weeks with and without hygromycin as a transfection stability assay. Flow cytometry results are represented as histograms. (**D**) Flow cytometry analysis of the expression of the integrins α_v_, β_5,_ and the α_v_β_5_ dimer in parental cells and clone 2. Results are presented as histograms (**left**) with the number indicating the percentage of clone 2 cells overexpressing α_v_β_5_ and a bar graph (**right**) showing the fold-change of integrin expression and the average ± SEM of the mean fluorescence intensity (MFI) of stained cells. Results were compared using a 1-way ANOVA with Tukey’s post hoc test. a.u., arbitrary unit.

After antibiotic selection, we used a western blot to analyze the expression of the exogenous integrins in whole lysates of 293T parental and modified cells. As expected, the exogenous integrin subunits were not detected in the parental cells (Prtl) but were detected in the pool of antibiotic-selected cells (Pool) (Figure 3A). To determine the percentage of cells overexpressing the integrin α_V_β_5_ in our multi-clonal pool, we used flow cytometry and found that most of the selected cells overexpress integrin α_V_β_5_, compared to parental cells. However, we observed two populations of 293T-α_V_β_5_^+^ cells: the majority of the pool (63%) had a lower overexpression population (×1.8 vs. Prtl), while 37% had a higher overexpression population (×10.9 vs. Prtl) (Figure 3B). We used a limited dilution from the pool to isolate α_V_β_5_^hi^ clones. We selected two clones in which we detected the expression of the exogenous integrins (Figure 3A). A flow cytometry analysis of these clones showed that clone 1 and clone 2 had a homogenous population with a high level of overexpression of α_V_β_5_ (Figure 3B). Then, we evaluated the stability of the transfection by culturing the cells with or without hygromycin for two weeks. While the cells from clone 1 started to have reduced α_V_β_5_ overexpression, the level of overexpression in clone 2 remained the same compared to cells cultured with hygromycin, indicating stability (Figure 3C). Lastly, we confirmed the levels of the α_V_ and β_5_ subunits, individually and as a dimer. Clone 2 cells had a six-fold increase in α_V_ and a four-fold increase in β_5_ subunits, and the α_V_β5 integrin level was four-fold higher when compared to parental 293T cells (Figure 3D). Considering the increased expression of the α_V_β_5_ integrin and the stability of the transfection, we chose to continue working with clone 2, now denominated 293T-α_V_β_5_^+^.

**Figure 4:**
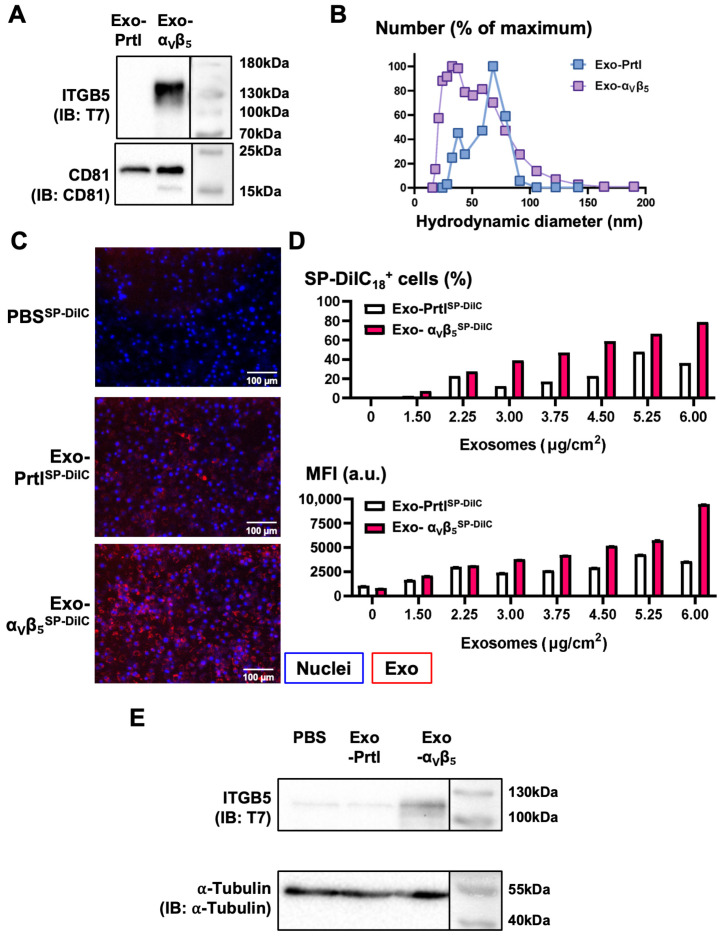
α_v_β_5_^+^ exosomes derived from 293T-α_v_β_5_^+^ cells carry the exogenous integrin and are internalized in RAW 264.7 macrophages in vitro. (**A**) Western blot analysis of the levels of the exogenous integrin subunit ITGB5-T7 in exosomes isolated from parental 293T cells (Exo-Prtl) and from 293T-α_v_β_5_^+^ cells (Exo-α_v_β_5_). CD81 was used as a loading control. (**B**) Measurement of the hydrodynamic diameter of the exosomes isolated from 293T cells, either parental (Exo-Prtl) or α_v_β_5_^+^ (Exo-α_v_β_5_). Results are presented as a histogram of the particle size distribution. (**C**) Fluorescence microscopy of RAW 264.7 cells incubated for 24 h with a control solution (PBS^SP-DiIC^) or SP-DiIC_18_-stained exosomes (3.0 µg/cm^2^, red) isolated from parental 293T cells (Exo-Prtl^SP-DiIC^) and from 293T-α_v_β_5_^+^ cells (Exo-α_v_β_5_^SP-DiIC^) (1.0 µM SP-DiIC_18_). Nuclei were stained with DAPI (blue). (**D**) RAW 264.7 cells were cultured for 24 h in the presence or absence of Exo-Prtl or Exo-α_v_β_5_ exosomes (1.5–6.0 µg/cm^2^) stained with SP-DiIC_18_ (1.0 µM) before analyzing cells by flow cytometry. Results are presented as the percentage of exosome-positive RAW 264.7 cells (upper bar graph) and the average ± SEM of the mean fluorescence intensity (MFI, a.u., bottom bar graph). (**E**) Western blot analysis of T7 tag (ITGB5) levels in cell lysates of RAW 264.7 cells incubated for 24 h with PBS, Exo-Prtl exosomes, or Exo-α_v_β_5_ exosomes (3.0 µg/cm^2^). α-tubulin was used as a loading control.

After successfully modifying 293T cells to overexpress α_V_β_5_, our next step was to characterize the exosomes derived from these modified cells. We isolated exosomes from parental and α_V_β_5_^+^ cells and evaluated the expression of the exogenous integrin using a western blot. We did not detect ITGB5-T7 in the exosomes of the parental cells, and it was only detected in the exosomes derived from α_V_β_5_^+^ cells (Figure 4A). Dynamic light scattering did not show any significant differences between the hydrodynamic diameter of the exosomes of the α_V_β_5_^+^ and the parental 293T cells (45.8 ± 22.7 nm vs. 59.5 ± 16.7 nm, respectively) (Figure 4B).

Next, we assessed whether the integrin could influence the internalization of exosomes in cultured macrophages. We added fluorescently labeled exosomes from parental 293T or 293T-α_V_β_5_^+^ cells to cultures of RAW 264.7 cells. After 24 h, fluorescence microscopy showed more exosome-positive cells when treated with α_V_β_5_^+^ exosomes than those treated with parental exosomes (Figure 4B). We then used flow cytometry to quantify these results. As we increased the amount of exosomes in the cultures, the percentage of exosome^+^ cells increased, with up to 80% of cells internalizing when 2 μg of exosomes was added (Figure 4D). The fluorescence intensity of exosome^+^ cells indicated a dose-dependent relationship with the amount of fluorescent exosomes internalized per cell (Figure 4D). In both cases, there was a marked increase in the uptake of α_V_β_5_^+^ exosomes compared to parental exosomes. Next, we asked if α_V_β_5_^+^ exosomes could transfer their cargo to RAW 264.7 cells. We prepared whole cell lysates from RAW 264.7 cells treated with parental or α_V_β_5_^+^ exosomes. The western blot analysis indicated the presence of ITGB5-T7 exclusively in RAW cells treated with α_V_β_5_^+^ exosomes (Figure 4E). Collectively, our findings indicate that α_V_β_5_ increases exosome internalization by macrophages, and α_V_β_5_^+^ exosomes transfer their cargo to cultured macrophages.

**Figure 5:**
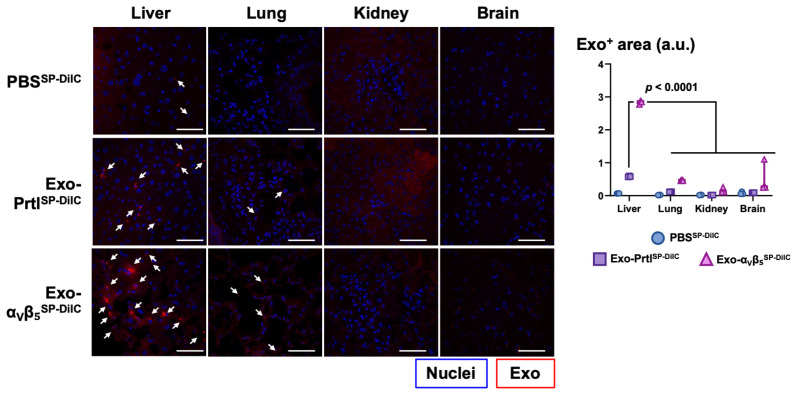
The overexpression of the integrin α_v_β_5_ increases exosome accumulation in the liver. Mice were inoculated with 40 µg of exosomes from parental 293T cells (Exo-Prtl^SP-DiIC^) or 293T-α_v_β_5_^+^ cells (Exo-α_v_β_5_^SP-DiIC^) stained with SP-DiIC_18_ (red) or with a control solution (PBS^SP-DiIC^) (5.0 µM SP-DiIC_18_). The next day, the liver, lungs, kidneys, and brain were collected, and cryosections with DAPI counterstaining (blue) were analyzed using a confocal microscope. Results are presented as (**left**) representative images of tissue sections and (**right**) the quantification of exosome-positive (Exo^+^) areas by fluorescence imaging in arbitrary units (a.u.) and were compared using a 2-way ANOVA with Tukey’s post hoc test. Scale bars indicate 50 µm. See also Supplemental Figure S1.

After confirming exosome internalization and cargo transfer in vitro, we sought to characterize the biodistribution of these exosomes in vivo. Balb/C mice were injected intravenously with PBS or fluorescent exosomes isolated from parental or α_V_β_5_^+^ 293T cells, and 24 h later, livers, lungs, brains, and kidneys were collected. Our observations of the mice during this period did not indicate changes in behavior or visible side effects (e.g., diarrhea). Confocal microscopy images of cryosections revealed minimal to no fluorescence signals in all the analyzed tissues of the mice treated with PBS (Figure 5 and Figure S1). When treated with parental exosomes, we observed fluorescent signals preferentially in the liver compared to the lungs, brain, and kidneys, which had 5.0, 29.2, and 6.5 times fewer exosomes, respectively (Figure 5 and Figure S1). Similarly, exosomes derived from 293T-α_V_β_5_^+^ cells preferentially accumulated in the liver rather than in the lungs, brain, or kidneys (with 6.2, 5.3, and 18.4 times fewer exosomes, respectively) (Figure 5 and Figure S1). More importantly, there were five times more exosomes in the livers of the mice inoculated with α_V_β_5_^+^ exosomes compared to the mice with exosomes from parental cells (Figure 5 and Figure S1). The distribution of the exosomes was uneven within the section, and some areas seemed to accumulate more exosomes than others. For technical reasons, we could not confirm whether the exosomes’ signals corresponded to Kupffer cells. These data demonstrate that the overexpression of the integrin α_V_β_5_ in 293T cells allows its transfer to their exosomes and enhances their accumulation in the liver in vivo.

### 3.3. Expression of shTgfb1 in 293T Cells Does Not Allow Its Exosomes to Knockdown Tgfb1 In Vitro

**Figure 6:**
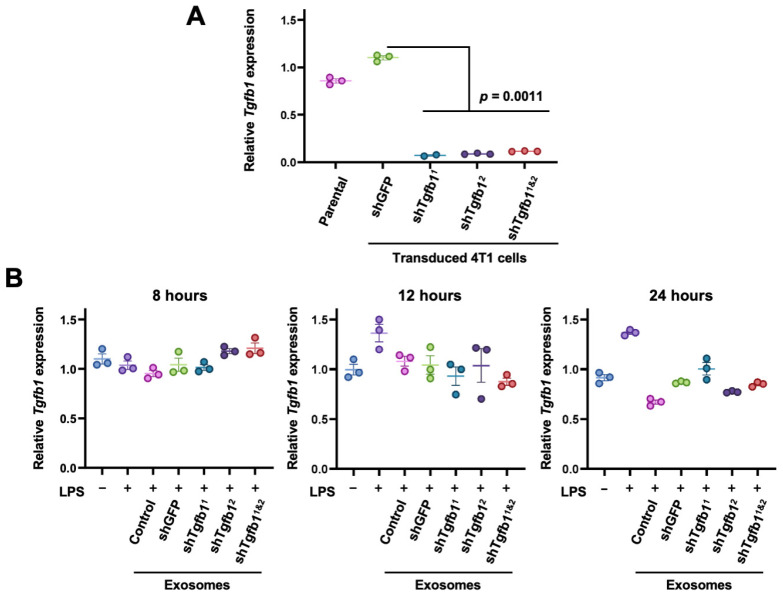
Exosomes from 293T cells expressing shTgfb1 do not knock down *Tgfb1* in RAW cells. 4T1 and 293T-α_V_β_5_ cells were transduced with pLKO.1-shGFP, pLKO.1-shTgfb1^1^, pLKO.1-shTgfb1^2^, or both shTgfb1 plasmids (shTgfb1^1&2^) and selected with puromycin. (**A**) After antibiotic selection, *Tgfb1* expression was evaluated by qRT-PCR in parental and transduced 4T1 cells with pLKO.1-shGFP, pLKO.1-shTgfb1^1^, pLKO.1-shTgfb1^2^, or both shTgfb1 plasmids (shTgfb1^1&2^), and results were compared using a 1-way ANOVA with Tukey’s post hoc test. (**B**) Exosomes derived from 293T-α_V_β_5_ (Control), 293T-α_V_β_5_-shGFP, 293T-α_V_β_5_-shTgfb1^1^, 293T-α_V_β_5_-shTgfb1^2^, or 293T-α_V_β_5_-shTgfb1^1&2^ were isolated and added to RAW 264.7 cultures for 8, 12, or 24 h, and *Tgfb1* expression was evaluated by qRT-PCR. Results were compared using a 1-way ANOVA with Tukey’s post hoc test. See also Supplemental Figure S2.

The secretion of TGF-β by Kupffer cells after the internalization of the exosomes derived from pancreatic cancer tumors is one of the initial events in the formation of a pre-metastatic niche in the liver [5]. To counter this, we sought to knock down *Tgfb1* by delivering shRNA against *Tgfb1* (shTgfb1) using our α_V_β_5_^+^ exosomes. We inserted different shTgfb1 sequences (shTgfb1^1^, GCTCTTGTGACAGCAAAGATA, or shTgfb1^2^, GTGGAACTCTACCAGAAATAT) in a pLKO.1 vector. To validate these shRNA against *Tgfb1*, we transduced mouse cells of the 4T1 breast cancer cell line with lentiviral particles. After antibiotic selection, we confirmed that shTgfb1^1^ or shTgfb1^2^, either alone or in combination, decreased *Tgfb1* by at least 90% compared to cells expressing shGFP (Figure 6A). The 293T-α_V_β_5_^+^ cells were then transduced to produce shTgfb1^1^, shTgfb1^2^, either alone or combined, or shGFP. The shRNA expression system was also functional since in 293T-α_V_β_5_^+^ cells expressing GFP, the shGFP construct silenced the expression of the GFP (Figure S2A). However, we could not determine the efficiency of the shTgfb1 construct against mouse *Tgfb1* in the human 293T-α_V_β_5_^+^ cells. Thus, to determine whether the transduced cells had integrated the lentiviral vector, we confirmed the expression of the puromycin resistance gene, puromycin *N*-Acetyltransferase (*pac*), in the transduced cells (Figure S2B). Hence, we isolated exosomes from transduced and non-transduced 293T-α_V_β_5_^+^ cells and added them to cultured mouse RAW 264.7 cells. However, the treatment with α_V_β_5_^+^shTgfb1^+^ exosomes did not affect the expression of *Tgfb1* after 8, 12, or 24 h of treatment (Figure 6B). These data indicate that the expression of shTgfb1 in 293T cells does not confer to their exosomes the capacity to knock down *Tgfb1* in vitro.

### 3.4. Overexpressed sBG-Myc mRNA Transfers to Exosomes and Allows for Partial Inhibition of TGF-β Signaling In Vitro

**Figure 7:**
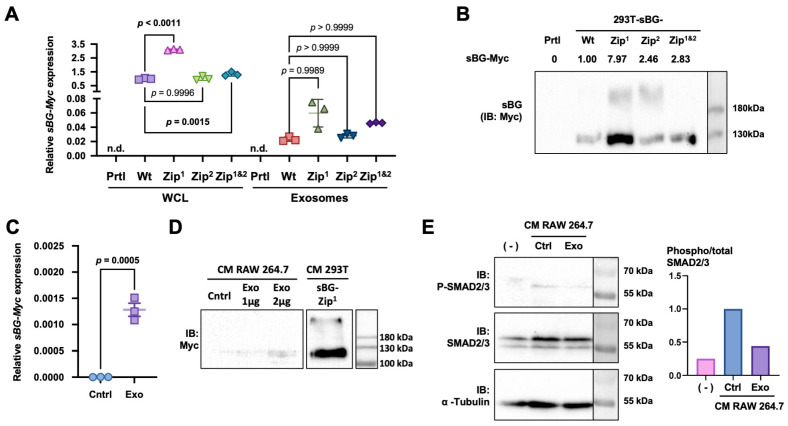
Exosome-mediated expression of soluble betaglycan decreases SMAD2/3 phosphorylation. 293T cells were transduced with pLJM1-sBG-Myc wild-type plasmid or pLJM1-sBG-Myc plasmid modified with exosome zipcode sequences in the 3′ UTR: Zip^1^, Zip^2^, and Zip^1&2^. (**A**) Evaluation of sBG mRNA expression by qRT-PCR in whole cell lysates (WCL) and exosomes from untransduced 293T cells (Prtl) or 293T cells transduced to express sBG-Myc, either wild type (WT) or with exosome zipcode sequences in the 3′ UTR: Zip^1^, Zip^2^, and Zip^1&2^. The results were compared using a 1-way ANOVA with Tukey’s post hoc test. (**B**) Western blot analysis of sBG-Myc in 293T (Prtl), 293T-sBG (Wt), 293T-sBG-Zip^1^, 293T-sBG-Zip^2^, and 293T-sBG-Zip^1&2^ cells in conditioned media. Numbers above membranes show quantitative analysis of Myc tag (sBG) expression. (**C**) RAW 264.7 cells were incubated for 24 h with 2 μg/cm^2^ of 293T-sBG-Zip^1^ cell-derived exosomes, and sBG mRNA expression was evaluated in treated cells using qRT-PCR and compared using an unpaired Student’s *t*-test. (**D**) Western blot analysis of the conditioned media of RAW 264.7 cells treated or not treated with 1 or 2 μg/cm^2^ of 293T-sBG-Zip^1^-cell-derived exosomes to detect the presence of sBG-Myc. The conditioned media of 293T-sBG-Zip^1^ cells were used as a positive control. (**E**) HepG2 cells were incubated for 24 h with complete EMEM media or the conditioned media of RAW 264.7 cells (Ctrl) or RAW cells treated with 2 μg/cm^2^ of exosomes derived from 293T-sBG-Zip^1^ cells. Results are presented as a Western blot (**left**) of phosphorylated and total SMAD2/3 (P-SMAD2/3 and SMAD2/3, respectively) and quantification (**right**) of the phospho/total SMAD2/3 ratio. α-tubulin was used as a loading control. See also Supplemental Figure S3.

Due to the apparent lack of efficiency of shRNA delivery by our exosomes, we sought to use a different mean to inhibit TGF-β. The soluble form of TGF-β type 3 receptor (TGFBR3) or betaglycan (sBG) acts as a TGF-β neutralizer, preventing the binding of TGF-β to the TGF-β type 2 receptor, hence inhibiting the activation of the TGF-β signaling pathway [7,17]. We proposed to overexpress sBG with an Myc tag at the C-terminus in 293T cells in order to be naturally loaded into their exosomes and to use them as a site-directed therapy. In addition, we sought to take advantage of a putative 25-nucleotide exosome zipcode, ACCCUGCCGCCUGGACUCCGCCUGU, identified in the 3′ UTR of mRNA enriched in exosomes [25], to increase the export of *sBG-Myc* mRNA to the exosomes. Using site-directed mutagenesis, we inserted one or two zipcode sequences downstream of the *sBG-Myc* coding sequence, denominated Zip^1^, Zip^2^, or Zip^1&2^. Following transduction and antibiotic selection, real-time PCR testing confirmed the expression of *sBG-Myc* in the transduced 293T cells, and a western blot analysis corroborated that the sBG-Myc protein translated from these mRNA was secreted in the conditioned media (Figure 7A,B). We isolated exosomes from the transduced cells (Figure S3A) and, using real-time PCR testing, detected *sBG-Myc* mRNA in the exosomes of all the transduced cells (Figure 7A). However, the mRNA levels appeared to be 30 to 50 times lower in the exosomes than in the whole cell lysates, and the addition of the exosome zipcodes did not increase the loading of these mRNA in the exosomes (Figure 7A). Although the levels of *sBG-Myc-Zip^1^* mRNA with the first zipcode were 2.6 times higher compared to those of the unmodified *sBG-Myc* (Wt) in exosomes, the difference was not statistically significant and was most likely due to the overall higher levels of mRNA of *sBG-Myc-Zip^1^* in the transduced cells (×3.1 vs. Wt, *p* < 0.0001) (Figure 7A). Due to these higher mRNA levels, we continued working with exosomes from 293T cells expressing *sBG-Myc-Zip^1^*. Dynamic light scattering confirmed these exosomes had a diameter of 39.9 ± 17.0 nm (Figure S3B), like the parental cells (Figure 1C), and they were well internalized by the RAW 264.7 cells (Figure S3C). When the RAW 264.7 cells were treated with exosomes isolated from the 293T-sBG-Myc-Zip^1^ cells, we detected the presence of sBG within the treated cells (Figure 7C). This led us to investigate the effect of sBG-Zip^1^ exosomes on the TGF-β signaling pathway.

As a pre-metastatic niche in vitro model, we used RAW 264.7 cells as surrogate Kupffer cells since they secrete TGF-β in the presence of LPS and cultured them in the presence or absence of exosomes from parental or sBG-Myc-Zip^1^ 293T cells. Their conditioned media were collected to treat HepG2 cells, a hepatic cell line, before testing for the phosphorylation of SMAD2/3. RAW cells were treated or not treated with exosomes from parental or sBG-Myc-Zip^1^ 293T cells. Compared to regular cell culture media, the conditioned media of RAW 264.7 cells treated with control exosomes increased SMAD2/3 phosphorylation in HepG2 cells (Figure 7D). This induction was partly reversed (−56%) when HepG2 cells were cultured with the conditioned media of RAW cells treated with the sBG-Myc-Zip^1^ exosomes, indicating an effective reduction in TGF-β signaling induction (Figure 7D). Overall, these data indicate that the overexpression of *sBG-Myc* in 293T cells can generate exosomes that transfer this mRNA to target cells that produce sBG protein to partly decrease TGF-β signaling in vitro. However, means to optimize the transfer of the *sBG-Myc* mRNA to the exosomes must be found to improve the efficiency of these nanocarriers.

## 4. Discussion

The pre-metastatic niche is set up from a distance by the primary tumor and increases the probability of colonization of distant organs by metastatic cells, the bottleneck of the metastatic cascade [26]. In the liver, it is triggered by elevated TGF-β production from Kupffer cells after the internalization of exosomes released by the primary tumor [5]. Although anti-TGF-β therapies have shown promising results in pre-clinical models, their application in clinical trials has resulted in significant adverse side effects. The dual role of TGF-β makes it difficult to target using systemic therapies. Nevertheless, these unwanted side effects could be mitigated through a site-directed approach using exosomes. These small extracellular vesicles can transport and deliver proteins and nucleic acids, while evading immune system clearance, which makes them highly attractive drug delivery systems. As described by Hoshino et al. [12], the distribution of exosomes throughout the body is not random, and different exosomes target specific organs. Integrins dictate exosome organotropism; for example, α_6_β_4_ directs the exosomes to the lungs and α_V_β_5_ to the liver.

Our study aimed to develop a liver-targeted anti-TGF-β treatment using functionalized α_V_β_5_^+^ exosomes. We chose 293T cells (also called HEK 293T) as a source of exosomes since they are expected to cause minimal adverse effects. Proteomic and transcriptomic analyses showed few disease-related or cancer-related proteins, mRNA, or miRNA present in these exosomes [20]. The small size of their exosomes (~50 nm) may enable them to evade phagocytosis by circulating monocytes, therefore extending their half-life in circulation [10]. Previous studies have shown that the treatment of mice with exosomes of 293T cells or from other human cell lines for up to 3 weeks did not cause toxicity or an immune response [12,27,28]. Finally, 293T cells have an endogenous expression of the integrin α_V_β_5_, making them a good candidate for our liver-specific approach. Since we inoculated the exosomes from the human 293T cells in mice, there might have been a risk that the human integrin α_V_β_5_ would not bind to the mouse fibronectin. However, the RGD motif that integrins bind to is conserved across species, and previously published work confirms that human cancer cells with a higher expression of α_V_β_5_ metastasize more to the liver [29]. In addition, the knockdown of β_5_ in human cancer cells decreased the accumulation of their exosomes in the liver [12], indicating that cross-species reactivity should not be a limitation for experiments in mice. In our in vivo experiments, fluorescently labeled exosomes were administered intravenously to mice and were detected in their liver 24 h post inoculation. To improve the transfer of these exosomes to the liver over other tissues, we modified the 293T cells genetically. Previously, the exosome surface has been modified to confer cell type targeting specificity [30]. The concept of generating site-specific exosomes was first developed by Alvarez-Erviti et al. [23], who engineered a membrane protein, the lysosome-associated membrane glycoprotein 2b (Lamp2b), fused to a neuron-specific rabies virus glycoprotein (RVG) peptide to target neuronal cells. Lamp2b fused with a targeting ligand is the most widely used method to direct exosomes to a specific cell type [30]. Our study proposes a novel and more straightforward approach to engineering exosomes to specifically target the liver, a method that has not yet been described. To increase exosome accumulation in the liver, we decided to overexpress α_V_β_5_. We co-transfected 293T cells with plasmids of α_V_β_5_^+^ exosomes coding the α_V_ and β_5_ subunits and obtained a subclone that stably overexpresses α_V_β_5_. When parental or α_V_β_5_^+^ exosomes were added to cultured macrophages, they demonstrated a higher level of internalization in RAW cells. In vivo, we confirmed that overexpressing α_V_β_5_ significantly improves the accumulation of the exosomes in the liver. Moreover, α_V_β_5_^+^ exosomes exhibited a preferential affinity for the liver over the lungs, kidneys, or brain, suggesting their potential use for liver-targeted therapy.

Exosomes can be loaded with a therapeutic agent (e.g., siRNA and chemotherapeutic drugs) using different methods, such as incubation, electroporation, or sonication [10,31,32]. However, these methods add additional steps to the exosome preparation, causing a decrease in the quantity of isolated exosomes, and the loading efficiency is not always optimal. Hence, we decided to modify the 293T cells to directly produce an anti-TGF-β agent that could be loaded in their exosomes for therapeutic purposes. Initially, we used two shRNA to knock down *Tgfb1*. Despite the efficacy of both shTgfb1 in reducing *Tgfb1* in transduced mouse cells, the exosomes derived from 293T cells expressing shTgfb1 failed to diminish *Tgfb1* levels in the treated macrophages in vitro. This lack of efficiency may stem from the internalization mechanism of the exosomes in the RAW cells. If they are phagocytosed and sorted into phagolysosomes [33], the shRNA could be degraded before being processed. Another limitation of this system is the difficulty in tracking the loading of the shRNA to the exosomes and their efficient transfer to the treated cells. Hence, we switched to an mRNA-based approach so that cells internalizing the exosomes could produce an anti-TGF-β protein. We transduced 293T cells to express mRNA encoding a soluble form of BG to neutralize TGF-β with an Myc tag at the C-terminus. *sBG-Myc* mRNA was detected in the 293T cells and their exosomes. However, these mRNA levels in the exosomes were 43 times lower than those in the cells. Two different parameters may restrict the amount of *sBG-Myc* mRNA in the exosomes of the 293T cells: the overall loading of the exosomes and the size of the mRNA for sBG-Myc.

Regarding mRNA size, the *sBG-Myc* mRNA transcript from the pLJM1 vector is about 4120 nt plus the polyA tail, which is double the average length of exosomal RNA (about 2100 nt) found by previous studies [34,35]. Others indicate that exosomes contain RNA that are less than 500 to 700 nt long [36,37]. This could indicate that instead of the full-length *sBG-Myc*, the exosomes could contain fragments of our mRNA of interest and explain the rather low level of protein produced by the RAW cells treated with our exosomes. Thus, it is possible that the mRNA expressed could be optimized to shorten it by coding only for the TGF-β binding domain of BG. Peinado et al. [4] found that the content of the exosomes is lower in normal cells when compared to cancer cells. Although we could switch to a model using modified cancer cells to produce exosomes, the other molecules loaded in their exosomes would likely contribute to establishing the pre-metastatic niche rather than preventing it; thus, we chose to use a non-cancerous cell line. Improving the loading of the exosomes could be achieved by engineering the 293T cells. The increased protein loading in extracellular vesicles was associated with the expression of RAB proteins that could be overexpressed in 293T cells [4]. The loading of RNA can also be improved by using sequences that target them in the exosomes. We added one or two sequences identified as exosome zipcodes in the 3′ UTR of the *sBG-Myc* mRNA, as they were found in mRNAs enriched in exosomes [25]. However, this did not allow for a specific increase in the amount of *sBG-Myc* mRNA in the exosomes of our 293T cells. The exosome loading mediated by this zipcode depends on the presence of and binding to miR-1289. Thus, it is possible that its level of expression in 293T cells restricts the efficacy of the exosome zipcode and that the overexpression of miR-1289 could serve as a means to compensate for this limitation and increase the levels of *sBG-Myc* in exosomes. Kojima et al. [38] devised an EXOsomal transfer into cells (EXOtic) device that allows for an improved delivery of mRNA in cell cytosol and loading into their exosomes, which could be a very efficient strategy. An alternative to mRNA would be to use miRNA to silence *TGFB1*/*Tgfb1* levels since EXOmotifs have been found that lead to the sorting and enrichment of these miRNA in exosomes [39]. Aside from natural loading within cells, it would be possible to switch back to extra-cellular loading, which has been demonstrated to be efficient too. Popowski et al. used electroporation to load exosomes with mRNA that were then lyophilized and prepared for inhalation delivery [40]. These improved loading strategies could also be combined with the enhancement of exosome production or release. Different proteins such as STEAP3 metalloreductase and syndecan-4 (SDC4) are involved in the formation of exosomes. The overexpression of both STEAP3 and SDC4, as well as a fragment of the L-aspartate oxidase (NadB), using a tricistronic system, caused a drastic increase in the release of exosomes from HEK293T cells [38]. Other systems have been developed to improve the packing of plasmids inside exosomes as well as the secretion of exosomes from cells, such as a cellular nanoporation biochip [41] or a nanoelectroporation system based on track-etched membranes (TM-nanoEP) [42], which could potentiate the production of functionalized α_V_β_5_^+^ exosomes.

Despite these limitations, treating RAW cells with our engineered exosomes allowed us to detect the mRNA *sBG-Myc* in RAW cells treated with these exosomes. More importantly, we detected low levels of sBG protein in the conditioned media of these RAW cells that partly reversed the induction of SMAD2/3 phosphorylation in hepatic cells in vitro. The activation of the TGF-β signaling pathway in stellate cells is crucial to the fibrotic response during the establishment of the pre-metastatic niche that enhances the development of liver metastases. Treatment with an inhibitor of TGFBR1 prevented this fibrotic reaction and decreased liver metastases in mice [5]. However, treatment with systemic inhibitors of TGF-β signaling has adverse effects on patients. In our study, we found that the α_V_β_5_^+^ exosomes could preferentially target the liver in vivo and then deliver anti-TGF-β agents such as *sBG-Myc* mRNA, with the potential to prevent side effects. In addition to treatment in patients at risk for liver metastases, these anti-TGF-β α_V_β_5_^+^ exosomes could also be used for the treatment of liver fibrosis caused by chronic hepatitis, alcohol abuse, or cirrhosis, in which TGF-β also drives the production of fibronectin and for which liver transplantation becomes the only therapeutic option [43].

## 5. Conclusions

This study sought to use the cargo-carrying capacity of exosomes and to take advantage of integrin-mediated organ specificity to deliver an anti-TGF-β agent to the liver. Through the overexpression of the integrin α_V_β_5_ in 293T cells, we generated exosomes that preferentially accumulate in the liver of mice after systemic administration. The expression of shRNA against *Tgfb1* in these cells did not allow its knockdown in treated cells. However, the expression of an mRNA coding for a soluble form of the TGF-β neutralizer betaglycan allowed for the isolation of exosomes that attenuated the TGF-β signaling pathway. While further research is needed to improve the transfer of these mRNA into the exosomes and determine their in vivo potential, the findings of this study provide a proof of concept that α_V_β_5_^+^ exosomes may serve as nanocarriers for liver-targeted therapies.

## Figures and Tables

**Table 1:** Sequences of oligonucleotides used for subcloning and colony PCR.

Name	Orientation	Sequence (5′ to 3′)
ITGB5	forward	CTCCATAGAAGACACCGACTCTAGACACCATGCCGCGGGCCCCGG
	reverse	TGTAATCCAGAGGTTGATTGTTAGCCCATCTGCTGGCCGCCGGTCATTGAAGCCATGTCGACGTCCACAGTGCCATTGTAGGATTTGTTGAACTTGTTGAAGGTG
Zip^1^	forward	CCTGGACTCCGCCTGTGCAGTATTCATCCACAATTTTAAAAG
	reverse	CGGCAGGGTGATCCTACATTTGTCTCGAGGTCGAG
Zip^2^	forward	GACTCCGCCTGTGAGATCCACTTTGGCCGC
	reverse	CAGGCGGCAGGGTTGCTGTCCCTGTAATAAACC
Zipcodeinsertion	forward	GAATTCTCGACCTCGAGACA
	reverse	AGGGCTGCCTTGGAAAAG
shTgfb1^1^	forward	GTATCTTTGCTGTCACAAGAGCTTTTTGAATTCTCGACCTCGAG
	reverse	TCGAGTATCTTTGCTGTCACAAGAGCCCGGTGTTTCGTCCTTTC
shTgfb1^2^	forward	GATATTTCTGGTAGAGTTCCACTTTTTGAATTCTCGACCTCGAG
	reverse	TCGAGATATTTCTGGTAGAGTTCCACCCGGTGTTTCGTCCTTTC

**Table 2:** Sequences of oligonucleotides used for real-time RT-PCR.

Gene	Organism	Gene ID	Orientation	Sequence (5′ to 3′)
*Tgfb1*	*Mus*	21803	forward	ACTGGAGTTGTACGGCAGTG
	*musculus*		reverse	GGGGCTGATCCCGTTGATTT
*Rpl32*	*Mus*	19951	forward	CAGGGTGCGGAGAAGGTTCAAGGG
	*musculus*		reverse	CTTAGAGGACACGTTGTGACAATC
*TGFBR3*	*Rattus*	29610	forward	ATACACAATGGCTCCCGTGG
	*norvegicus*		reverse	GGAAGAGTGGGTTGTCCAGG
*RPL32*	*Homo*	6161	forward	CAGGGTTCGTAGAAGATTCAAGGG
	*sapiens*		reverse	CTTGGAGGAAACATGTGAGCGATC
*pac*			forward	ATGACCGAGTACAAGCCCAC
			reverse	ACACCTTGCCGATGTCGAG

## Data Availability

The raw data supporting the conclusions of this article will be made available by the authors on request.

## Supplementary Materials

The following supporting information can be downloaded at https://www.mdpi.com/article/10.3390/biology13121066/s1, Figure S1: The overexpression of the integrin αvβ5 increases exosome accumulation in the liver; Figure S2: Transduction of 293T-α_V_β_5_ cells for expression of shRNA against *GFP* or *Tgfb1*; Figure S3: Characterization of exosomes from 293T-α_V_β_5_ cells transduced to express *sBG-Myc* mRNA.
