# Supplementary material for: Development of Liver-Targeting αVβ5+ Exosomes as Anti-TGF-β Nanocarriers for the Treatment of the Pre-Metastatic Niche
Source: Biology (Basel). 2024 Dec 19;13(12):1066. doi: 10.3390/biology13121066 (PMC11673512; doi:10.3390/biology13121066)
Supplement: Supplementary file 1 [file biology-13-01066-s001.zip › biology-3336327-supplementary.pdf]

Supplemental information

Development of Liver-Targeting $\alpha_v\beta_5^+$ Exosomes as anti-TGF-β Nanocarriers for the Treatment of the Pre-Metastatic Niche

Paloma Acosta Montaño, Eréndira Olvera Félix, Veronica Castro Flores,
Arturo Hernández García, Ruben D. Cadena-Nava, Octavio Galindo Hernández,
Patricia Juárez, and Pierrick GJ. Fournier

Supplemental information - Table of contents

- **Supplemental Figure S1.** The overexpression of the integrin $\alpha_v\beta_5$ increases exosome accumulation in the liver.
- **Supplemental Figure S2.** Transduction of 293T-$\alpha_v\beta_5$ cells for expression of shRNA against *GFP* or *Tgfb1*.
- **Supplemental Figure S3.** Characterization of exosomes from 293T-$\alpha_v\beta_5$ cells transduced to express *sBG-Myc* mRNA.

Supplemental Figure S1A. The overexpression of the integrin $\alpha_v\beta_5$ increases exosome accumulation in the liver.

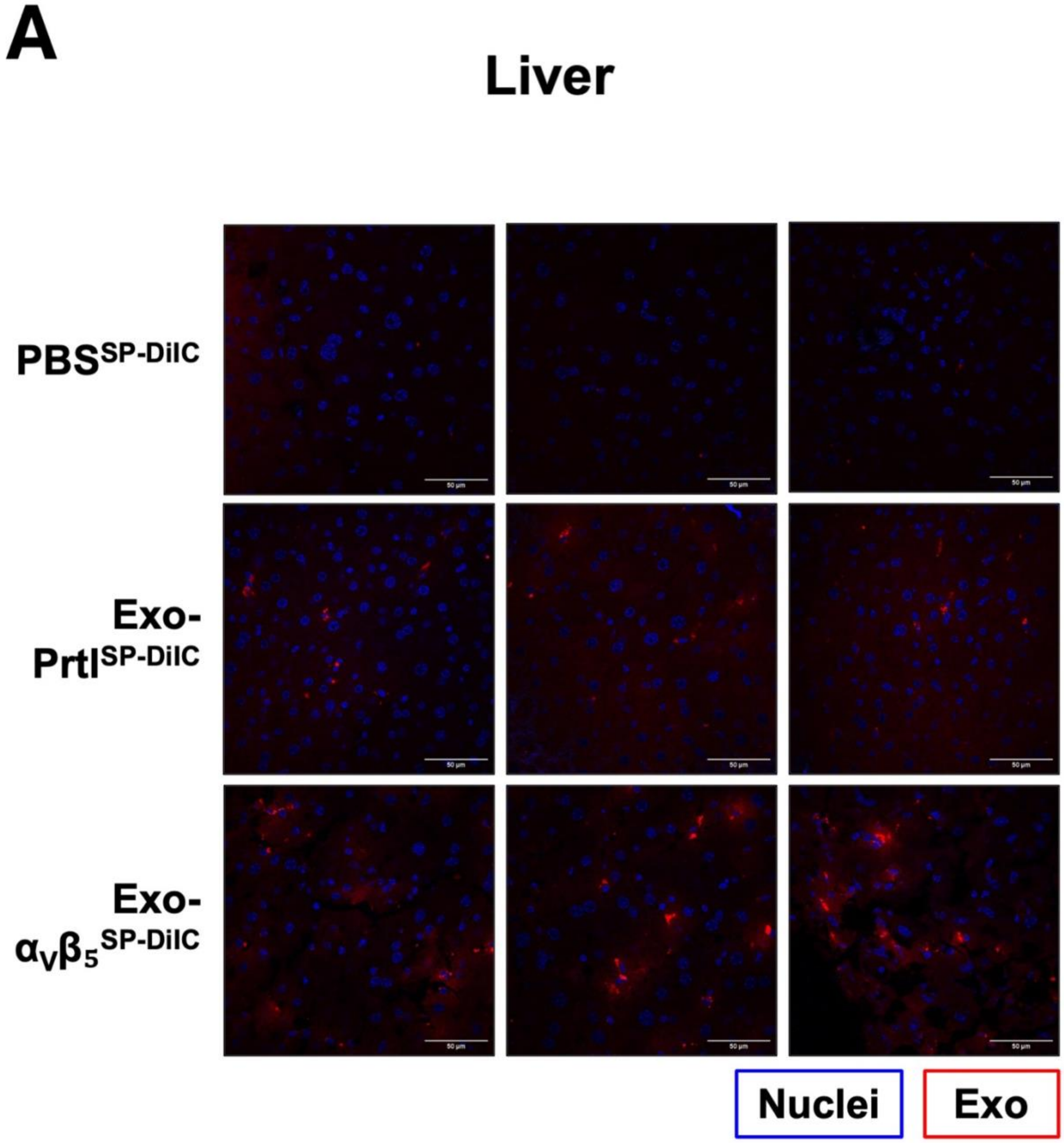

Supplemental Figure S1B. The overexpression of the integrin $\alpha_V\beta_5$ increases exosome accumulation in the liver.

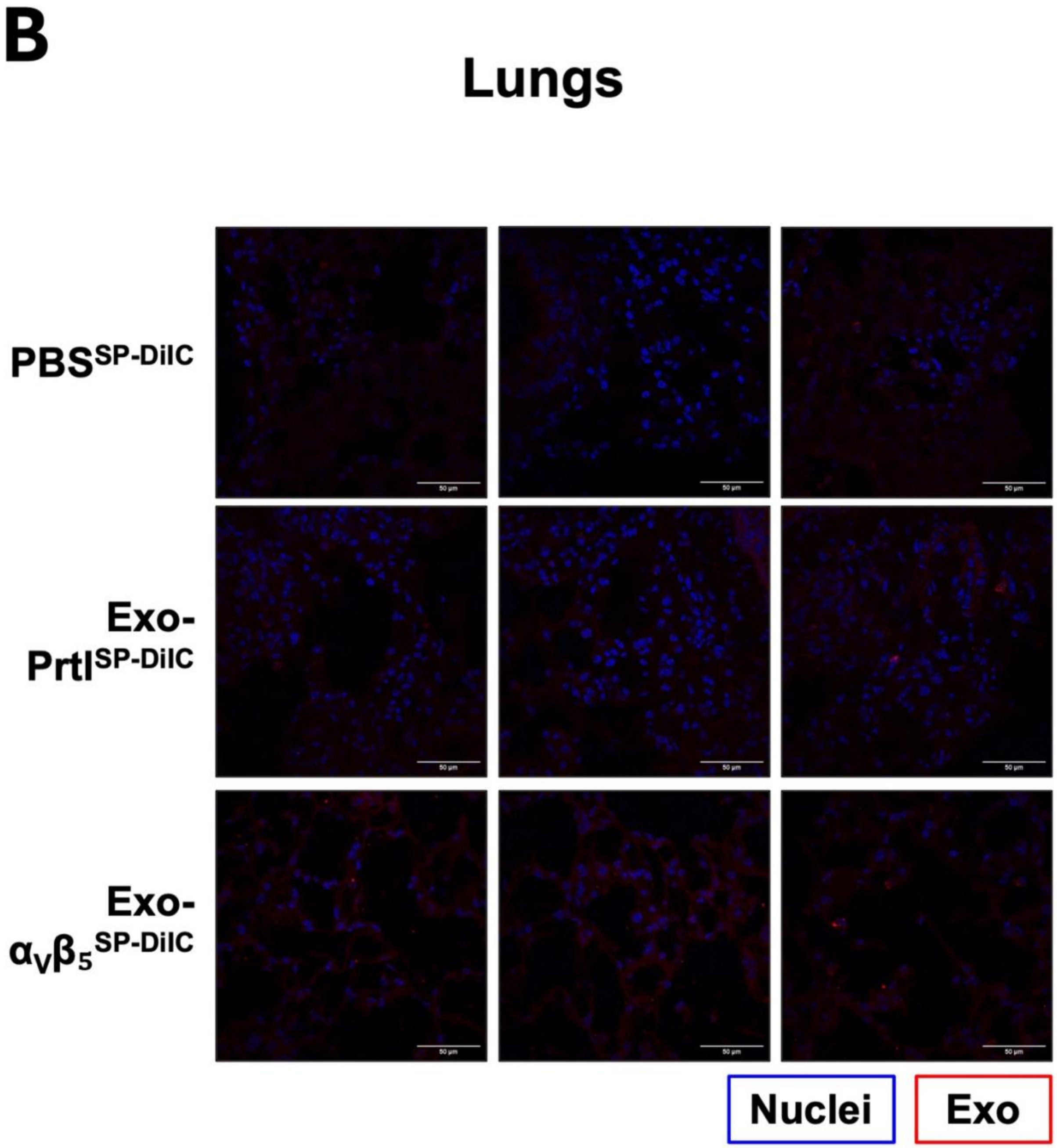

Supplemental Figure S1C. The overexpression of the integrin $\alpha_v\beta_5$ increases exosome accumulation in the liver.

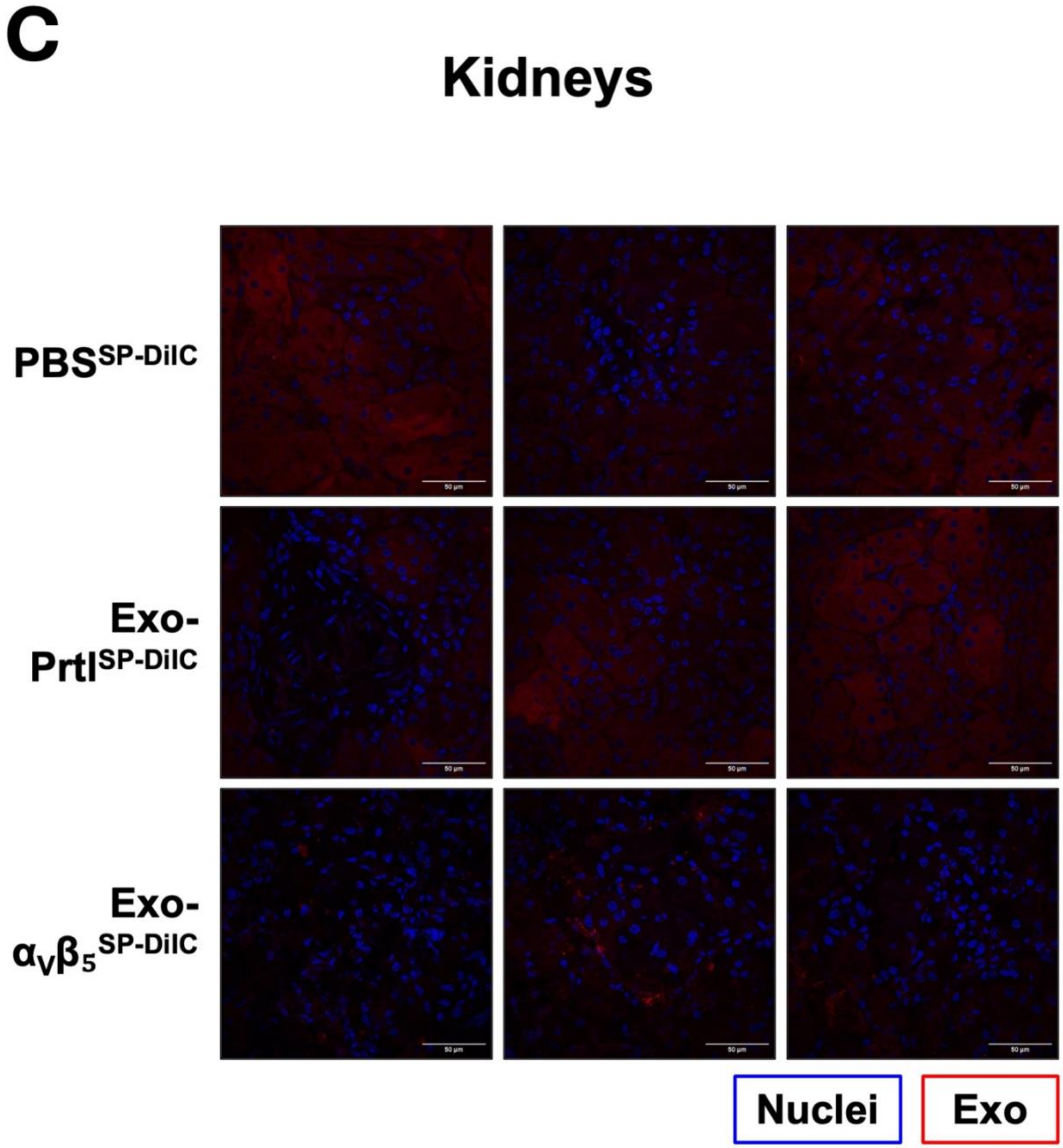

Supplemental Figure S1D. The overexpression of the integrin $\alpha_v\beta_5$ increases exosome accumulation in the liver.

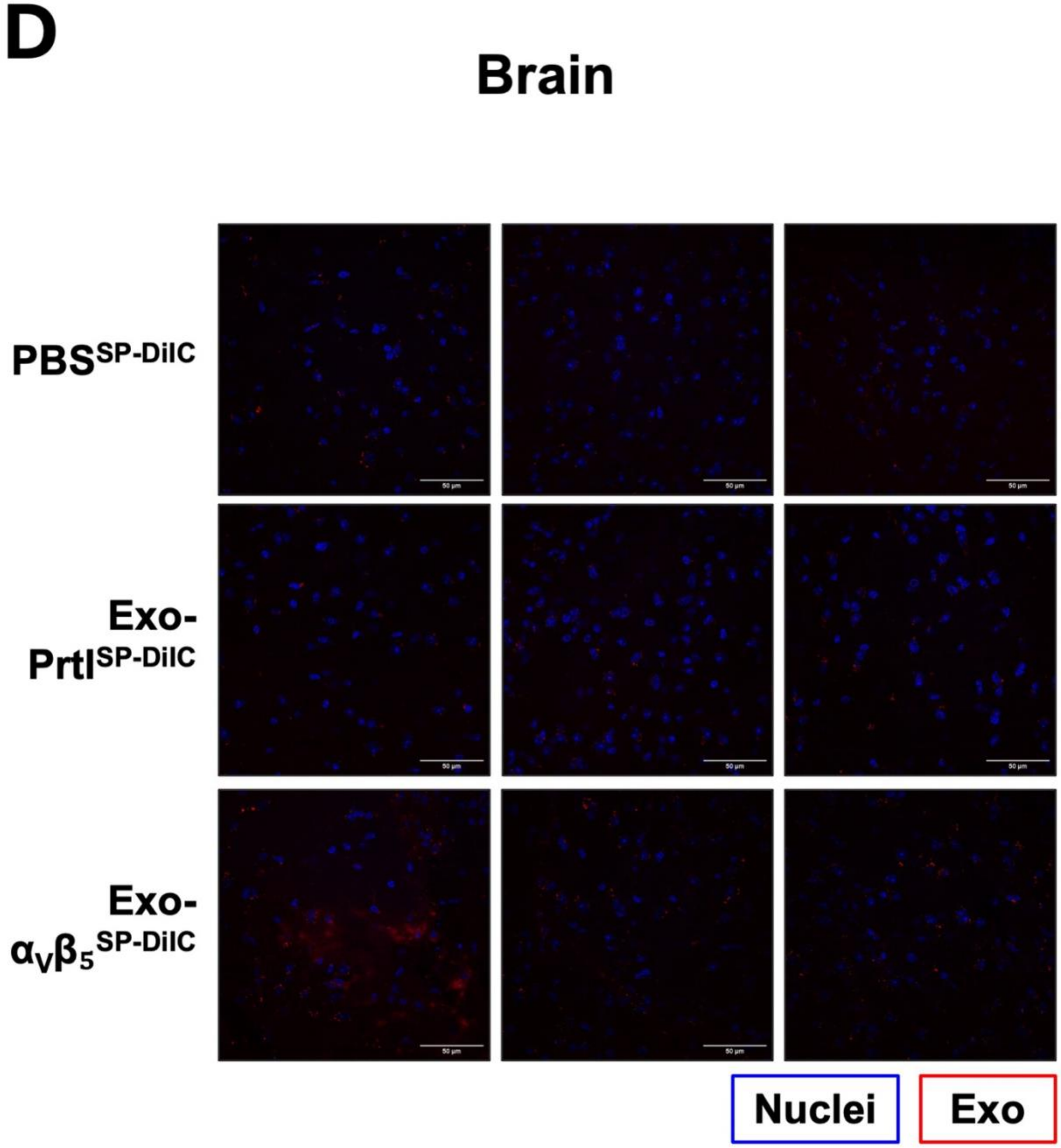

Supplemental Figure S1. The overexpression of the integrin $\alpha_v\beta_5$ increases exosome accumulation in the liver. Mice were inoculated with 40 μg of exosomes from parental 293T cells (Exo-Prtl$^{SP\text{-}DiIC}$) or 293T-$\alpha_v\beta_5^+$ cells (Exo-$\alpha_v\beta_5^{SP\text{-}DiIC}$) stained with SP-DiIC$_{18}$ (red) or with a control solution (PBS$^{SP\text{-}DiIC}$) (5.0 μM SP-DiIC$_{18}$). The next day, the liver, lungs, kidneys, and brain were collected, and cryosections with DAPI counterstaining (blue) were analyzed using a confocal microscope. Results are presented as three images of different areas of the tissue sections of **(A)** liver, **(B)** lungs, **(C)** kidneys, and **(D)** brain where fluorescence was detected. The scale bar indicates 50μm.

Supplemental Figure S2. Transduction of 293T-$\alpha v\beta_5$ cells for expression of shRNA against *GFP* or *Tgfb1*.

A

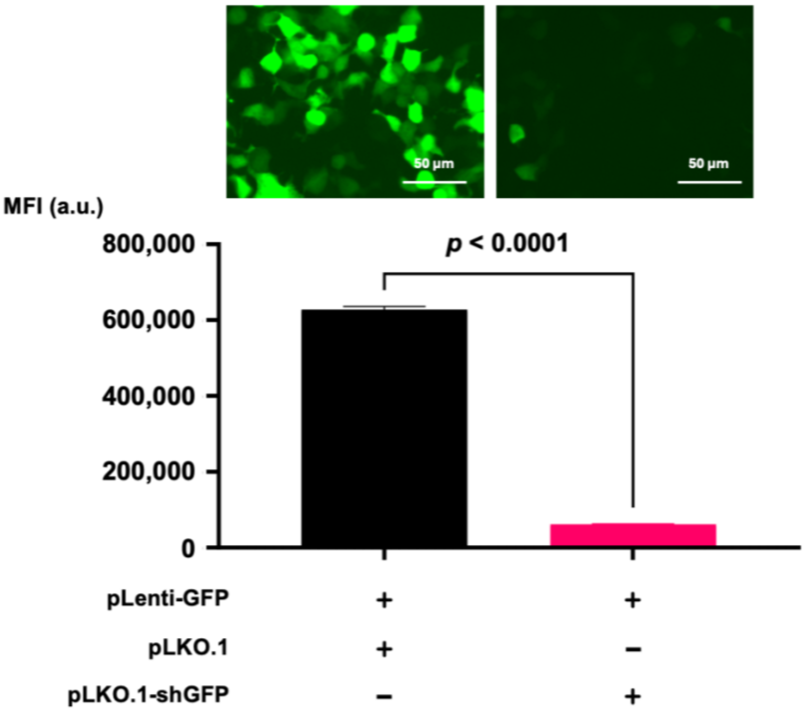

B

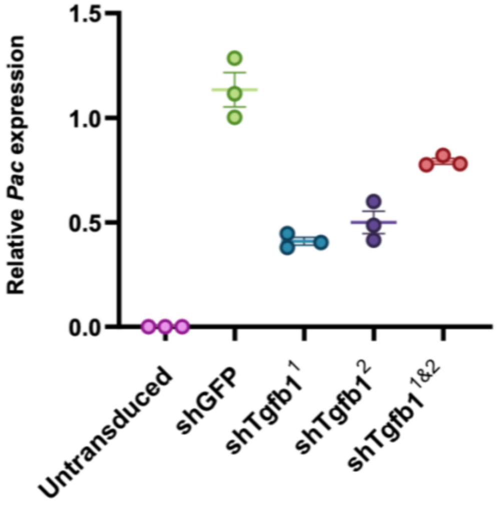

Supplemental Figure S2. Transduction of 293T-$\alpha_v\beta_5$ cells for expression of shRNA against GFP or *Tgfb1*. **(A)** 293T-$\alpha_v\beta_5$ cells were co-transfected with the pLenti-CMV-GFP-Puro plasmid for GFP expression and the pLKO.1-puro or pLKO.1-shGFP. Twenty-four hours later, cells were observed under a fluorescence microscope (*upper images*) and analyzed in a flow cytometer to measure the mean fluorescence intensity (MFI) (*lower panel*). a.u., arbitrary unit. **(B)** 293T-$\alpha_v\beta_5$ cells were transduced with pLKO.1-shGFP, pLKO.1-shTgfb1^{1}, pLKO.1-shTgfb1^{2}, or both shTgfb1 plasmids (shTgfb1$^{1\&2}$) and selected with puromycin. After antibiotic selection, *Tgfb1* expression was evaluated by qRT-PCR in parental and transduced 293T-$\alpha_v\beta_5$ cells.

Supplemental Figure S3. Characterization of exosomes from 293T-$\alpha v\beta_5$ cells transduced to express *sBG-Myc* mRNA.

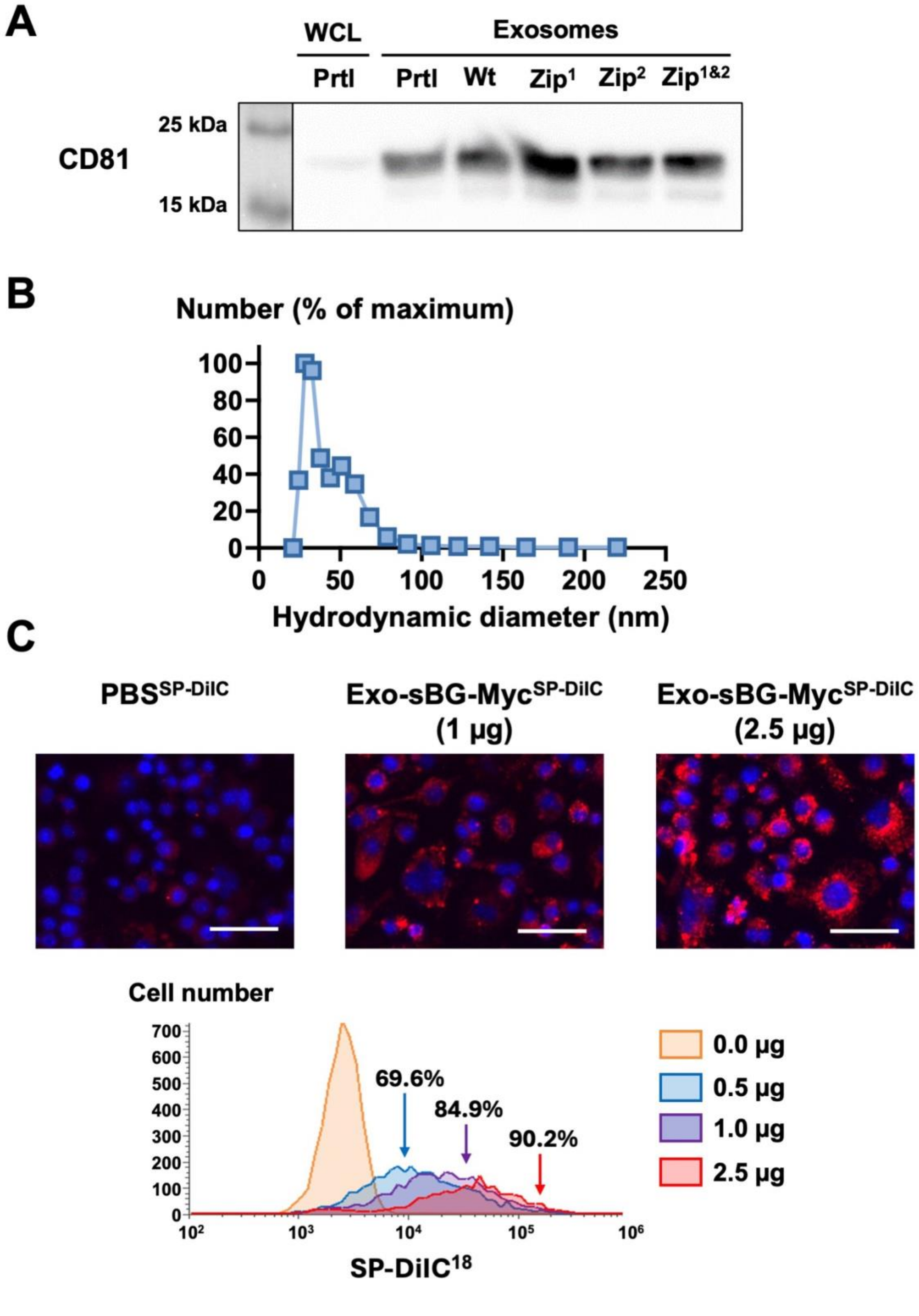

Supplemental Figure S3. Characterization of exosomes from 293T-$\alpha_v\beta_5$ cells transduced to express *sBG-Myc* mRNA. **(A)** 293T cells were transduced with pLJM1-sBG-Myc wild-type plasmid or pLJM1-sBG-Myc plasmid modified with exosome zipcode sequences in the 3′ UTR: Zip^1, Zip^2, and $Zip^{1\&2}$. After antibiotic selection, exosomes were isolated and levels of CD81 marker were assessed by western blot. WCL, whole cell lysate. Prtl, parental cells. **(B)** Measurement of the hydrodynamic diameter of the exosomes isolated from 293T-sBG-Zip^1 cells. Results are presented as a histogram of the particle size distribution. **(C)** RAW 264.7 cells were cultured for 24 hours with a control solution ($PBS^{SP\text{-}DiIC}$) or SP-$DiIC_{18}$-stained exosomes (1.0 or 2.5 μg/cm^2, red) isolated from 293T-sBG-Zip^1 cells (Exo-sBG-$Myc^{SP\text{-}DiIC}$) before analyzing the cells by (*upper panel*, the scale bar indicate 50 μM) or flow cytometry (*lower panel*, percentages indicate the amount of SP-$DiIC_{18}^+$ cells).
